# Fabrication of Antimicrobial Peptide-Loaded PLGA/Chitosan Composite Microspheres for Long-Acting Bacterial Resistance

**DOI:** 10.3390/molecules22101637

**Published:** 2017-09-29

**Authors:** Yuanyuan Li, Rongwei Na, Xiumei Wang, Huiying Liu, Lingyun Zhao, Xiaodan Sun, Guowu Ma, Fuzhai Cui

**Affiliations:** 1 State Key Laboratory of New Ceramic & Fine Processing, School of Materials Science and Engineering, Tsinghua University, Beijing 100084, China; yuanyuan0154@163.com (Y.L.); narongwei@126.com (R.N.); lyzhao@mail.tsinghua.edu.cn (L.Z.); sunxiaodan@mail.tsinghua.edu.cn (X.S.); cuifz@mail.tsinghua.edu.cn (F.C.); 2 Department of Prosthodontics, School of Stomatology, Dalian Medical University, Dalian 116044, China; mgw64024@163.com; 3 Department of Stomatology, Shengli Oil Field Central Hospital, Dongying 257034, China

**Keywords:** antimicrobial peptides, electrospraying, bacterial resistance, PLGA, composite microspheres

## Abstract

An antimicrobial decapeptide, KSL-W (KKVVFWVKFK-CONH_2_), which could maintain stable antimicrobial activity in saliva, has therefore been widely used to inhibit biofilm formation on teeth and prevent the growth of oral microorganisms for related infectious diseases treatment. In order to control the release of KSL-W for long-term bacterial resistance, KSL-W-loaded PLGA/chitosan composite microspheres (KSL/PLGA/CS MSs) were prepared by electrospraying and combined crosslinking-emulsion methods. Different formulations of microspheres were characterized as to surface morphology, size distribution, encapsulation efficiency, in vitro drug release, and antimicrobial activity. Antibacterial experiment demonstrated the prolonged antimicrobial and inhibitory effects of KSL/PLGA/CS MSs on oral bacteria. Moreover, the cell proliferation assay proved that the released KSL-W antibacterial dosage had no cytotoxicity to the growth of osteoblast MC3T3-E1. Thus, our study suggested that the KSL-W-loaded PLGA/CS composite microspheres may have potentially therapeutic applications as an effective drug delivery system in the treatment of oral infectious diseases such as periodontitis and periodontitis, and also within bone graft substitutes for alveolar bone augmentation.

## 1. Introduction

Oral infections, generally caused by bacteria and viruses, are very common diseases in the oral and maxillofacial regions including dental caries, periodontitis, gingivitis, oral mucosal infections, peri-implantitis, etc., which may be very mild or lead to serious problems [1]. For example, peri-implantitis, a type of destructive inflammatory process surrounding dental implants that can give rise to progressive loss of alveolar bone around the implant, is considered one of the main causes of dental implant failure [1,2,3]. Peri-implantitis is associated with the formation of a bacterial biofilm and compromised immunity at the implant/tissue interface. Microorganisms such as *Fusobacterium nucleatum* (*F.n.*) accumulate in the subgingival crevice around dental implants and play a significant role in accelerating the co-aggregation of pathogens, promoting the formation of biofilm, and thus inducing the development of peri-implantitis [2,3,4]. Therefore, antibiotic treatments and the maintenance of antibacterial microenvironments are of vital importance for oral health.

The antibiotic treatments with conventional antibiotic drugs such as ampicillin, vancomycin, and gentamycine have become more and more complicated by the emergence of multi-drug resistant bacteria. Antimicrobial peptides (AMPs), since their initial discovery in the 1980s, have been demonstrated as a promising alternative to conventional antibiotics [5]. AMPs represent a class of short cationic amphiphilic peptides presented ubiquitously in natural organisms as the innate immune system with a broad spectrum of activity against bacteria, viruses, fungi, and even transformed or cancerous cells [5,6,7,8]. Not like most of antibiotics that act on specific proteins, AMPs aim at bacterial membranes and subsequently disintegrate bacterial cell membranes to overcome the problem of antibiotic resistance and the microbial gene mutation. According to these advantages, AMPs have therefore been widely studied as alternatives to traditional antibiotics in recent years.

KSL (KKVVFKVKFK-CONH_2_) is a novel antimicrobial decapeptide that is developed through synthetic combinatorial library technology showing a wide range of activity against microbial pathogens [9,10]. Previous studies have showed that KSL was stable in artificial saliva and acidic buffer, and could effectively inhibit the growth of oral biofilm and prevent the development of oral bacterial pathogens [11,12,13]. Also, KSL-W (KKVVFWVKFK-CONH_2_), the analogue of KSL, was proved to display improved stability in saliva and simulated gastric conditions with preserved antimicrobial activity [11]. At the same time, KSL-W exhibits no cytotoxicity on host cells. Therefore, KSL-W is also widely accepted for effectively killing bacteria and controlling biofilm formation in oral applications [14].

The site-specific delivery and controlled release of therapeutic drugs, bioactive proteins, and peptides have been attracting more and more attention in the field of biomaterials and drug delivery. Encapsulation in the form of nanoparticles or microspheres is considered an effective drug delivery method for proteins/peptides to protect their bioactivities, enhance the therapeutic efficiency, and reduce drug side-effects as well. In this study, prolonged release of KSL-W to the targeted diseased area is thought to be imperative to maintain long-term antimicrobial effect for satisfying infection control. Poly(lactide-*co*-glycolide) (PLGA) microspheres have been extensively applied as drug delivery system because of their good biocompatibility and adjustable biodegradability [15,16]. However, the locally acidic micro-milieu associated with PLGA hydrolysis may result in protein aggregation and denaturation, and thus confine its applications [17,18]. Therefore, chitosan (CS), a positively charged linear polysaccharide, has been used with PLGA to neutralize the acidic degradation product of PLGA [19,20,21,22]. Chitosan is a biodegradable natural polymer with low toxicity, ideal biocompatibility, and excellent hemostatic and antimicrobial properties, which makes it suitable for many kinds of biomedical applications. Wang et al. successfully developed PLGA/CS composite microspheres through the emulsion method for the long-term delivery of oligopeptides derived from BMP-2 [21,22]. In our study, similar PLGA/CS microspheres were fabricated and studied for the sustained release of KSL-W.

In previous studies, PLGA microspheres were generally produced by phase separation, emulsification/solvent-extraction, or emulsification/solvent-evaporation [23,24]. However, from a technical standpoint, it is difficult to achieve desirable microspheres with uniform size distribution, high drug encapsulation, and good loading efficiency because smaller molecules can hardly enter PLGA microspheres sufficiently via conventional preparation methods. The electrospraying technique is a useful technique to prepare microspheres, embedding bioactive proteins through ejecting a polymer solution under high voltage power and ventilation conditions [25,26]. In addition, it is very easy to use electrospraying to completely control the drug encapsulation and size distribution of microspheres [26].

In this paper, sphere-in-sphere PLGA/CS composite microspheres were fabricated by electrospraying and combined crosslinking-emulsion methods for increased encapsulation efficiency and prolonged antimicrobial peptide release.

## 2. Results

### 2.1. Morphology and Structure Characterizations of KSL-W-Loaded Microspheres

**Figure 1:**
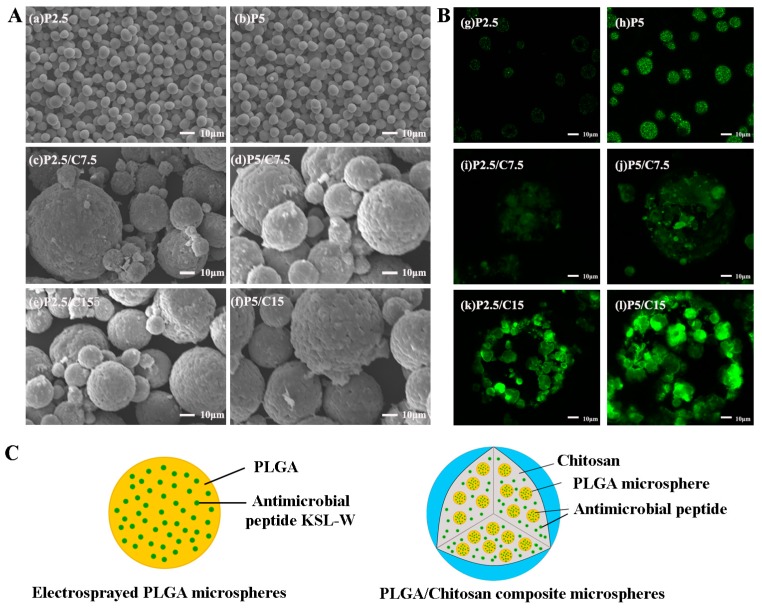
(**A**) The typical scanning electron microscope (SEM) morphologies of KSL-W-loaded PLGA and PLGA/CS composite microspheres; (**B**) Visualized distributions of FITC-conjugated KSL-W in PLGA and PLGA/CS microspheres under laser scanning confocal microscope (LSCM); (**C**) A schematic diagram of a KSL-loaded PLGA microsphere and a KSL-loaded PLGA/CS microsphere. PLGA, poly(lactide-co-glycolide); CS, Chitosan; FITC, fluorescein isothiocyanate.

The typical SEM morphologies of PLGA microspheres and PLGA/CS composite microspheres loaded with different masses of KSL-W are shown in Figure 1. It can be seen that all the formulations of microspheres were spherical and dispersed well. The electrosprayed PLGA microspheres for the two formulations exhibited smooth surfaces and quite uniform particle diameters of around 7 μm (Figure 1Aa,Ab). Furthermore, the PLGA/CS microspheres showed no obvious morphological differences among the four formulations with compact and corrugated surfaces and similar size distributions (Figure 1Ac–Af). Encapsulation of antimicrobial peptides did not change the surface morphology nor the particle size of the PLGA and PLGA/CS microspheres dramatically.

In order to confirm the encapsulation of KSL-W within the microspheres, fluorescein isothiocyanate (FITC)-conjugated KSL-W peptides were used to visualize the distribution under a Laser Scanning Confocal Microscope. As shown in Figure 1B, all the microspheres (MSs) had positive fluorescence emissions, indicating the successful encapsulation of peptides within the microspheres. Figure 1Bg,Bh indicated KSL-W peptides distributed evenly in the PLGA microspheres, displaying dose-dependent florescence intensity. Figure 1Bi–Bl demonstrated the encapsulations of not only KSL-W peptides but also the PLGA microspheres enwrapped by chitosan shells, forming PLGA/CS composite microspheres. While it could be noted that the KSL-W peptides dispersed in PLGA/CS microspheres not as homogeneous as those in PLGA microspheres, they are enriched in the surfaces of PLGA microspheres. The reason for this may be the electrostatic repulsion between positive charges of KSL-W and chitosan, as well as the electrostatic attraction of KSL-W with negative charges of PLGA.

### 2.2. The Size Analysis of the Microspheres

**Figure 2:**
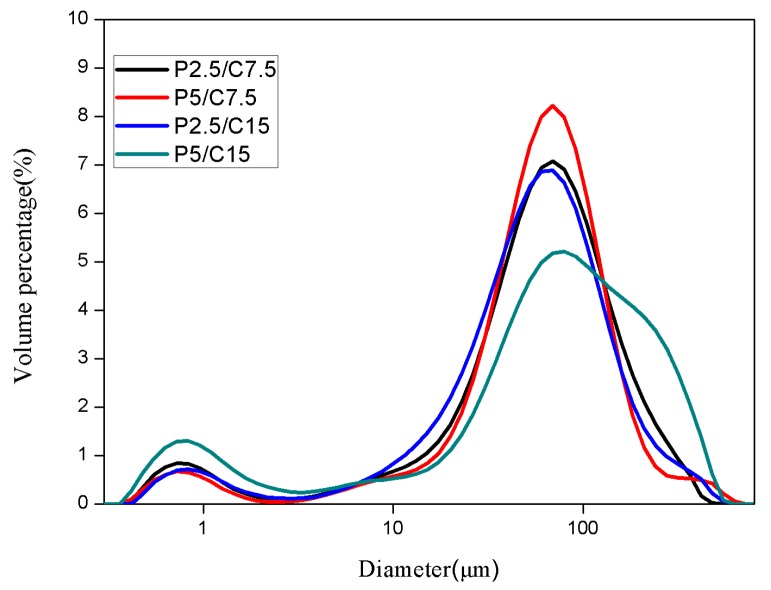
Size distribution of KSL-W-loaded PLGA and PLGA/CS composite microspheres.

A laser particle size analyzer was then used to determine the particle size distribution quantitatively, as shown in Figure 2. The average particle size as determined from the laser particle size analyzer was 61.14 ± 4.44 μm for formulation P2.5/C7.5, 66.54 ± 4.74 μm for formulation P5/C7.5, 67.06 ± 5.08 μm for formulation P2.5/C15, and 79.91 ± 5.01 μm for formulation P5/C15, as listed in Table 1. The size of PLGA/CS MSs increased a little bit with the increase of the total invested mass of KSL-W.

### 2.3. The Encapsulation Efficiency of the PLGA/CS Composite Microsphere

The KSL-W encapsulation behavior in the microspheres was characterized as the encapsulation efficiency (EE) and drug-loading rate (DLR), which are listed in Table 1. The PLGA microspheres fabricated by the emulsion electrospraying process had extremely high EE of over 90%. Also, the EE of KSL-W in the PLGA/CS microspheres prepared by the emulsification method could reach 60–70%. Although the higher the amount of the KSL-W added during the fabrication, the higher the encapsulation efficiency could be obtained, there were no significant differences between the EEs among different formulations of PLGA/CS microspheres. The drug-loading rates of PLGA/CS microspheres are in the range of 1–3% due to the low molecular weight of KSL-W. The more KSL-W administrated, the higher the drug-loading rate obtained under the same conditions.

### 2.4. In Vitro Release Profile of KSL-W from PLGA/CS Microspheres

**Figure 3:**
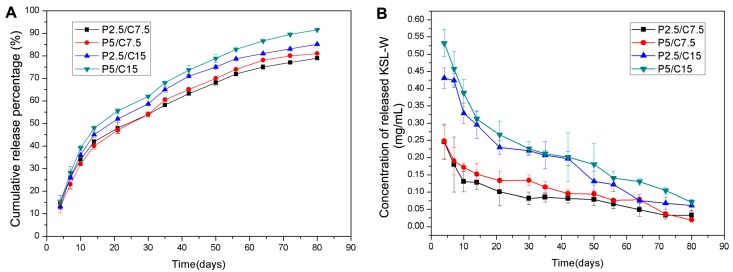
In vitro release profile of KSL-W from PLGA/CS microspheres. (**A**) Cumulative release percentage of KSL-W; (**B**) The concentration of released KSL-W. (Data are shown as the means ± standard deviation, *n* = 3).

The in vitro release profile of KSL-W from PLGA/CS microspheres suspended in phosphate buffered solution (PBS) is described in Figure 3. As shown in Figure 3A, all formulations of the PLGA/CS composite microspheres showed a similar release curve trend with rapid release (about 25–35%) in the first 10 days and a successive sustained release behavior up to 80 days. Around 45–60%, 70–80%, and 80–90% KSL-W peptides were released cumulatively from the composite microspheres after 30, 50, and 80 days of incubation, respectively. The concentration of released KSL-W was measured and is shown in Figure 3B. After 80 days of incubation and drug release, the concentration of KSL-W was about 0.02 mg/mL, which is still higher than the minimum bacterial-inhibitory concentration of KSL-W (0.0156 mg/mL) [27].

### 2.5. The Stability Assessment of the KSL-W

**Figure 4:**
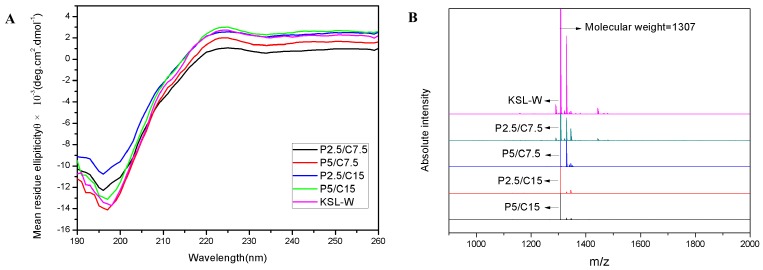
The stability assessment of KSL-W. (**A**) Far-UV CD spectra and (**B**) Mass spectrometry spectra of the KSL-W peptides before and after encapsulations.

In order to testify the structural stability of the released KSL-W, the secondary structure and relative molecular weight of KSL-W used was measured with Far-UV circular dichroism (CD) and mass spectrometry. Far-UV CD spectra of KSL-W peptides before and after encapsulations are shown in Figure 4A. The CD spectra of KSL-W released from P2.5/C7.5, P5/C7.5, P2.5/C15, and P5/C15 are similar to the CD spectrum of the original KSL-W. In these curves, there was a negative peak at 198 nm and a small and broad positive peak at 220 nm attributed to the random coil conformation of KSL-W. Mass spectrometry spectra of the original KSL-W and the KSL-W released from P2.5/C7.5, P5/C7.5, P2.5/C15, and P5/C15 are shown in Figure 4B. The relative molecular weights of KSL-W were obtained from the peaks of the spectra. In comparison to original KSL-W, it can be seen that the relative molecular weight of the released KSL-W remained at 1307, which is consistent with its theoretical value. The retention of the secondary structure and relative molecular weights indicated that no degradation or aggregation happened to KSL-W throughout the procedures of encapsulation, storage, and release, which was a key prerequisite for peptide KSL-W to present bioactive functions accurately.

### 2.6. The Biocompatibility of Peptide KSL-W

**Figure 5:**
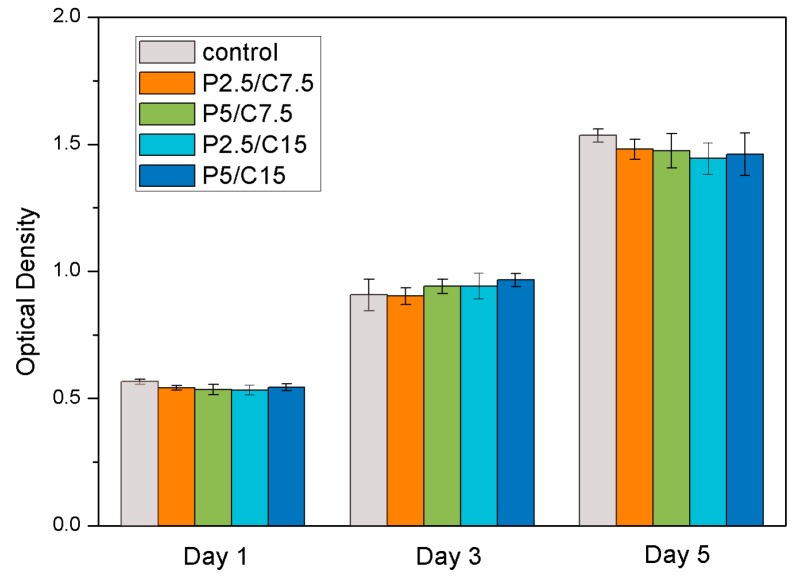
CCK-8 assay of MC3T3-E1 proliferation cultured in the conditioned medium containing different concentrations of KSL-W. (Data are shown as the means ± standard deviation, *n* = 4).

In order to evaluate the potential cytotoxicity of the antimicrobial peptide KSL-W on host tissue cells, CCK-8 assay was used to quantitatively examine the cell proliferation of MC3T3-E1 cultured in the conditioned medium containing different concentrations of KSL-W, as shown in Figure 5. Cell growth and proliferation showed no significant differences (*p* > 0.05) between the control group (cell culture in the regular growth medium) and the test groups (cell culture in four different formulations of conditioned media). The results indicate that the released KSL-W and the degradation products of PLGA/CS composite microspheres did not affect the viability or cellular metabolism, which ensured the safety of the further potential applications of KSL-W-loaded PLGA/CS composite microspheres.

### 2.7. The Antibacterial Assay

**Figure 6:**
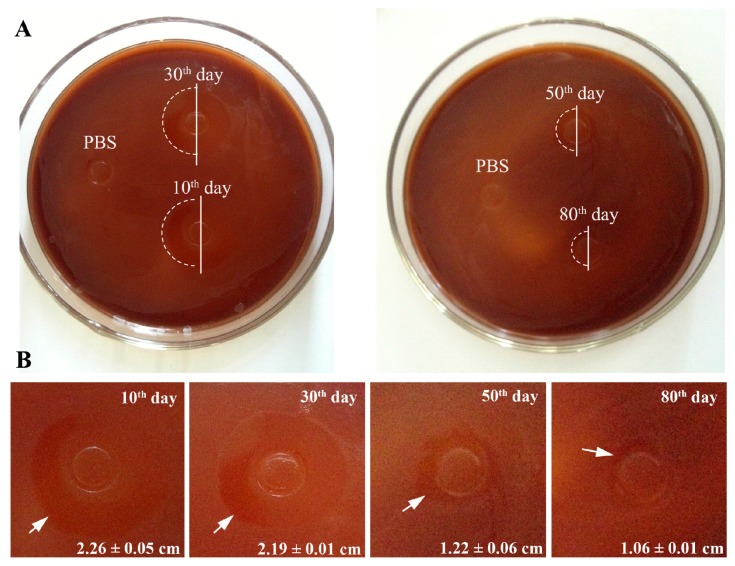
The typical morphologies of the inhibition zone induced by the extract solutions of KSL-W-loaded PLGA/CS microspheres after three days of incubation on agar plate. (**A**) Gross images of the agar plates; (**B**) High-magnification images of the inhibition zones. The edges of the inhibition zones are indicated by white arrows. The diameter of the inhibition zone was calculated (*n* = 3).

The antibacterial activity of the extract solutions against *F.n.* was evaluated by the inhibition zone assay. As shown in Figure 6, the typical morphologies of inhibition zones were clearly observed on the agar plate surrounding the Oxford cups, in which the extract solutions of KSL-W-loaded PLGA/CS microspheres from different time intervals of 10 days, 30 days, 50 days, and 80 days were loaded, while no such inhibition zone was observed in the PBS control group. The most obvious inhibition zone with a diameter of 2.26 cm was seen in the group with 10 day-extract solution, indicating the superior antibacterial activity of the KSL-W-loaded PLGA/CS microspheres in the early stage of the releasing progress. Along with the time extension, the inhibition zone appeared to be small and indistinct. The diameter of the inhibition zone in the test group with 80 day-extract solution was about 1.06 cm.

## 3. Discussion

In this study, the PLGA/CS composite microspheres exhibited an excellent release profile for the sustained release of KSL-W in a controllable manner. The release profile of drug-loaded microspheres is affected by the properties of the MSs such as the drug/polymer mass ratio, the physicochemical properties of the drug and polymer, and the particle size. The PLGA microspheres fabricated by the emulsion electrospraying process had smooth surfaces and quite uniform particle diameters of around 7 μm, which made them able to be encapsulated in CS shells more evenly in a sphere-in-sphere structural pattern, probably favoring better drug release behavior. Moreover, the electrosprayed PLGA microspheres could reach much higher encapsulation efficiency of over 90% than the microspheres fabricated by the W/O/W emulsion-solvent evaporation method. Within the initial 10 days, the PLGA/CS MSs underwent a quick release period with about 30% KSL-W released. This phenomenon was mainly due to the easier and faster release of KSL-W distributed near microsphere surfaces in a diffusion-controlled mode [26]. After that, the PLGA/CS MSs released about 60% KSL-W in the following 70 days in both diffusion- and degradation-controlled fashions. As a result, the initially quick release of the KSL-W contributed to resist acute inflammation, and the sustained release for up to 80 days from the PLGA/CS composite microspheres benefitted the maintenance of an antibacterial microenvironment. Moreover, all the formulations of PLGA/CS MSs showed similar drug release trends, which indicated that the concentration of released KSL-W could be easily adjusted by the amount initially added in PLGA and CS solutions. The more KSL-W added, the larger the released concentration.

Recently, AMPs have attracted increasing interest as an alternative to common antibiotics in biomedical applications against infections, mainly because of their broad-spectrum antibacterial activity and little bacterial resistance. Most AMPs are characterized by a positive net charge and hydrophobic amino acids that make them able to insert into and disintegrate negatively-charged bacterial cell membranes. However, as is well-known, most mammalian cells are also negatively charged; therefore, the long-acting antibacterial effect and the biosafety of AMPs on mammalian cells are also thought to be key issues for clinical applications. Some previous studies indicated that cationic AMPs present a certain level of cytotoxic and hemolytic activity to human cells and erythrocytes, respectively. Other studies showed that AMPs have an essential property to interact preferentially with bacteria compared to mammalian cells, due to the specific biophysical and biochemical properties of bacterial membranes. Therefore, the AMPs concentration in the local milieu should be accurately controlled to provoke both effective antibacterial activity and minimal cytotoxicity to ensure the health of mammalian cells.

In our studies, PLGA/CS composite microspheres were fabricated for the controlled release of KSL-W. The mouse preosteoblast cell line, MC3T3-E1, was then used to evaluate the cytocompatibility of the released AMPs, indicating that the initially high concentration of AMPs released from all of the formulations of microspheres exhibited no cytotoxicity. At the same time, the KSL-W-loaded PLGA/CS MSs showed long-term antibacterial activity for up to 80 days. *F.n.*, evaluated here, is an early colonizer in the subgingival crevice around dental implants and plays a significant role in accelerating the co-aggregation of pathogens, promoting the formation of biofilm, and thus inducing the development of peri-implantitis. The minimum inhibitory concentration against *F.n.* reported in the previous study is about 0.0156 mg/mL [27]. Yet, the minimum concentration of KSL-W solution after 80 days of incubation in our study was about 0.0190 mg/mL, which was still higher than the minimum inhibitory concentration reported previously, showing effective antibiotic activity. A series of fabrication processes from encapsulation, and storage, to release did not destroy the antibacterial activity of KSL-W. Therefore, our results indicate that the sustained release system of AMPs based on PLGA/CS MSs have potential applications for the treatment of peri-implantitis and other oral diseases.

## 4. Materials and Methods

### 4.1. Chemicals

Antimicrobial peptide KSL-W (KKVVFWVKFK-CONH_2_, Mw: 1307) was custom-synthesized by Qiangyao Bio-Technology Co., Ltd. (purity > 90%, Shanghai, China). Poly (lactic-*co*-glycolic acid) (PLGA, 75:25, Mw: 5.0 × 10^4^) was purchased from the Medical Equipment Research Institute (Jinan, China). Chitosan (Mw: 1 × 10^5^–3 × 10^5^) was purchased from the Bailingwei Science and Technology Co., Ltd. (Beijing, China). All other chemicals used were of analytical grade and obtained from Chemical Reagent Co., Ltd. (Beijing, China).

### 4.2. Preparation of PLGA Microspheres

KSL-W-loaded PLGA microspheres were prepared through emulsion electrospraying. The PLGA was dissolved in trichloromethane (CHCl_3_) at a concentration of 60 mg/mL. Different amounts of KSL-W (2.5 mg or 5.0 mg) were dissolved in 50 μL of deionized water and then added to 1 mL of the PLGA solution to form a W/O emulsion by sonication on ice with an ultrasonic crasher (Scientz-IID, Ningbo Science Biotechnology Co., Ltd., Ningbo, China) at a power of 300 W for 20 s. The emulsion solutions were then electrosprayed under an ambient atmosphere. The emulsion solutions were placed in a 1 mL syringe and passed through a blunt stainless-steel nozzle (inner diameter = 0.34 mm) at a constant flow rate of 1 mL/h using a micro-infusion pump. The applied voltage to the nozzle tip was 5.23 kV, and the distance between the nozzle tip to the aluminum collection plate was 20 cm. The organic solvent was removed during the electrospraying process by evaporation, leaving solid microspheres on the collection plate. The PLGA microspheres were then freeze-dried for 24 h to remove the residual solvent. Two formulations of KSL-W-loaded PLGA microspheres with different amounts of added peptide were abbreviated as P2.5 and P5.0, respectively.

### 4.3. Preparation of PLGA/CS Composite Microspheres

KSL-W-loaded PLGA/CS composite microspheres were prepared with the crosslinking emulsification method. First of all, the 2% aqueous acetic acid solution was prepared and used as the solvent to dissolve chitosan at a concentration of 3% (*w*/*v*). Then, 30 mg of different formulations of PLGA microspheres were added to 10 mL of 3% CS solution, respectively, whilst stirred magnetically at room temperature, during which different amounts of KSL-W solutions (7.5 mg, 15.0 mg, 7.5 mg, and 15.0 mg) were also added to the PLGA/CS mixture. After that, the PLGA/CS/KSL-W mixture was poured into a mixture of 60 mL liquid paraffin and 1.8 mL Span 80 to form the W/O emulsion, which was then stirred magnetically for 30 min at room temperature. We then dropped 5 mL of 5% (*w*/*v*) aqueous sodium tripolyphosphate (TPP) solution slowly into the emulsion while stirring mildly for an additional 3 h. Afterwards, the resulting emulsion was settled overnight, forming solid microspheres. The final PLGA/CS/KSL-W microspheres were washed three times with excess amounts of petroleum ether and isopropyl alcohol, and then freeze-dried for 24 h to remove the residual solvent [19]. According to different amounts of peptides added within the PLGA and CS microspheres, the sequential formulations of products were abbreviated as P2.5/C7.5, P5/C7.5, P2.5/C15, and P5/C15.

### 4.4. Morphology Characterization of KSL-W-Loaded Microspheres

Scanning electron microscopy (JSM-7001F, JEOL Ltd., Tokyo, Japan) was used to characterize the surface morphology of microspheres. KSL-W-loaded PLGA microspheres and PLGA/CS microspheres were dispersed in water and ethyl alcohol, respectively, and then dropped on the surface of a metallic sample stand with a bi-adhesive carbon tape. After being dried under an ambient atmosphere, the samples were sputter-coated with gold and examined at an accelerating voltage of 15 kV.

In order to trace the distribution of KSL-W in the microspheres, FITC-conjugated KSL-W peptides were encapsulated within the microspheres and then examined using a Laser Scanning Confocal Microscope (Zeiss LSM710, excitation wavelength of 488 nm, Carl Zeiss Ltd, Oberkochen, Germany).

The size distribution of PLGA microspheres and PLGA/CS microspheres were determined using a laser particle size analyzer (Mastersizer 2000, Malvern Instruments Ltd., Worcestershire, UK). The PLGA/CS microspheres were dispersed in water.

### 4.5. Characterization of KSL-W Encapsulation Behavior

The KSL-W encapsulation behavior in the microspheres was characterized as the encapsulation efficiency (EE) and drug-loading rate (DLR), which were defined using the following equations:

$$\mathrm{Encapsulation}\;\mathrm{efficiency}\;\left(\%\right)\;=\left(\frac{\text{Amount of KSL-W in microspheres}}{\text{Amount of KSL-W added initially}}\right)\times \;100$$

$$\mathrm{Drug-loading rate}\;\left(\%\right)\;=\left(\frac{\text{Amount of KSL-W loaed in microspheres}}{\text{Total weight of KSL-W loaded microspheres}}\right)\times 100$$

For determining the amount of KSL-W loaded in PLGA microspheres, 10 mg of KSL-W-loaded PLGA microspheres were dissolved by adding 1 mL of acetonitrile and 3 mL of 0.01 M hydrochloric acid solution with shaking for 1 h in a wrist-action shaker. The extracted KSL-W in the supernatant was obtained after centrifugation. In order to calculate the total amount of KSL-W loaded in PLGA/CS microspheres, KSL-W was extracted from the microspheres through the following steps. First, 10 mg of PLGA/CS microspheres was dispersed in 2 mL of 2% aqueous acetic acid solution to dissolve the external chitosan thoroughly. After centrifugation, the supernatant (S1) was collected and the precipitate was dried and weighed. The precipitate was further dissolved by adding 0.7 mL acetonitrile and 1.3 mL 0.01 M of hydrochloric acid solution while stirring. After centrifugation, the supernatant (S2) was obtained. S1 and S2 were detected by a UV-VIS spectrophotometer to calculate the total mass of KSL-W within PLGA/CS MSs, which was marked as M1 for the mass of KSL-W in S1 and M2 for that in S2. The mass of KSL-W was calculated according to the extracted KSL-W solution concentration that was determined by UV-VIS spectrophotometer at 280 nm (UV759S, Shanghai Precision & Scientific Instrument Co., Ltd., Shanghai, China). The standard curve was plotted using the standard KSL-W solutions with given concentrations.

### 4.6. In Vitro Drug Release Profile

The in vitro release profile of KSL-W from PLGA/CS microspheres suspended in PBS was evaluated for up to 80 days. Briefly, 100 mg of KSL-W-loaded PLGA/CS microspheres were added into a 5 mL centrifuge tube containing 1 mL PBS (pH = 7.4), which was placed in a shaking bath (Model THZ-C, Taicang Laboratorial Equipment Factory in China) at 60 rpm and 37 °C. The microsphere suspensions were centrifuged at 5000 rpm for 10 min to retrieve the supernatants and replaced with 1 mL of fresh PBS at different intervals (4, 7, 10, 14, 21, 30, 35, 42, 50, 56, 64, 72, and 80 days). The aforementioned UV-VIS spectrophotometer (UV759S) method was used to determine the KSL-W concentrations in the released supernatant. Each formulation was examined in triplicate for preliminary statistical analysis.

### 4.7. The Stability Assessment of the KSL-W

The structural stability of peptide KSL-W after encapsulation and release was assessed by the secondary structure and molecular weight.

The secondary structure of the KSL-W released from composite microspheres after 30 days of incubation was monitored by Far-UV circular dichroism (CD, J-715-150L, JASCO, Tokyo, Japan). Released KSL-W solution was analyzed at room temperature in a quartz cuvette with a path length of 0.1 cm and a scanning speed of 100 nm/min in a wavelength range of 190–260 nm.

Matrix-assisted laser desorption/ionization time-of-flight Mass Spectrometry (MALDI-TOF-MS, Autoflext, Bruker Daltonic Inc., Bremen, Germany) was used to monitor the molecular weights of the KSL-W released in the supernatant. Briefly, 2 μL of 4-hydroxy-cinnamic acids and 2 μL of released supernatant were mixed uniformly. Then, the resultant mixture was dried at room temperature for 1 μL. A nitrogen gas laser further dissociated the samples under the positive ion reflector mode. Lastly, we collected and analyzed the peak signals (*m*/*z*) of the samples.

### 4.8. The Biocompatibility of Peptide KSL-W

The biocompatibility of PLGA/CS composite microspheres loaded with different masses of KSL-W was assessed using CCK-8. MC3T3-E1 cells as representative cells were cultured in a dish containing α-MEM supplemented with 10% (*v*/*v*) fetal bovine serum (FBS, Life Technologies, Inc., Rockville, MD, USA), 100 IU/mL penicillin, and 100 IU/mL streptomycin. In order to obtain the drug containing extract, 10 mg each of the composite microspheres was incubated with α-MEM supplemented for a 10-day period at 37 °C. Then, 1 × 10^4^ MC3T3-E1 cells per well were cultured in a humidified incubator with 5% CO_2_ atmosphere at 37 °C. After 24 h, cells were incubated with the drug extract. Fresh drug extract was replaced every two days and the CCK-8 assay was carried out after one, three, and five days. At each time point, the medium was removed, and fresh medium with CCK-8 (media:CCK-8 = 10:1) was added in each well for 3 h of incubation before measurement with a microplate reader (Bio-Rad, Model680, Bio-Rad Laboratories, Inc., Hercules, CA, USA) at a wavelength of 450 nm. Four repeated measurements for each group were carried out for statistical analysis.

### 4.9. The Antibacterial Assay

*F.n.* was used in this study to evaluate the antibacterial activity of the KSL-W-loaded PLGA microspheres. *F.n.* (ATCC 10953) was cultured in a liquid Bacto-tryptone-yeast extract-ascorbic acid-glucose 5 mg/mL NaCl solution, and incubated at 37 °C under anaerobic conditions (80% N_2_, 10% CO_2_, and 10% H_2_) for 48 h. Then, the bacterial cells were harvested by centrifugation at 10,000 rpm for 10 min, washed with sterile phosphate-buffered saline (PBS), and diluted to 1 × 10^8^ CFU/mL by using a spectrophotometer-based standard curve calculation. The inhibition zone assay was performed using the agar diffusion method (Oxford cup method). Briefly, to determine the inhibition zone, bacterial cells were uniformly distributed on the surface of the blood agar plate (Bacto-tryptone-yeast extract-ascorbic acid-agar, with fresh defibrous sheep blood for more nutrition), and then the Oxford cups with a diameter of 6 mm were placed onto the agar plates. After incubating at 37 °C for half an hour, 0.25 mL of released liquid isolated from P2.5/C7.5 at different time intervals of 10 days, 30 days, 50 days, or 80 days was added into the Oxford cup. PBS served as a negative control. The inhibition zones forming around the cylinders were measured after three days of incubation.

### 4.10. Statistical Analysis

All numerical data are reported as the means ± standard deviation. The statistical analysis was performed with one-way analysis of variance and Student’s *t*-test. The data were considered statistically significant when *p* < 0.05. All of the data were analyzed using SPSS 13.0 software for Windows, Student Version (IBM Corporation, Armonk, NY, USA).

## 5. Conclusions

In this study, different formulations of KSL-W-loaded PLGA/CS composite microspheres were prepared by electrospraying and combined crosslinking-emulsion methods. The PLGA/CS MSs had a sphere-in-sphere structure with the PLGA microspheres embedded in the external chitosan shells. The PLGA/CS composite microspheres showed high encapsulation efficiency and long-term sustained release of KSL-W up to 80 days. The structural integrity and molecular weight of the antimicrobial peptides in the process of encapsulation was not changed through the analysis of the Far-UV circular dichroism spectrum and MALDI-TOF-MS. Moreover, the antibacterial assay indicated that the KSL-W released from PLGA/CS composite microspheres retained long-term antibacterial activity against *F.n.* Our study suggested that KSL-W-loaded PLGA/CS composite microspheres may be applied as an effective drug delivery system in the treatment of oral infectious diseases as well as within bone graft substitutes for alveolar bone augmentation.

## Figures and Tables

**Table 1:** The mean diameter and encapsulation efficiency of the PLGA/CS composite microspheres.

MSs Formulations	KSL-W Mass (mg)			Mean Diameter (µm) *	Drug-loading Rate (DLR) (%) **	Encapsulation Efficiency (EE) (%) **
	M1	M2	Total			
P2.5	2.5	—	2.5	6.82 ± 0.71	3.72 ± 0.21	93.00 ± 5.28
P5	5.0	—	5.0	6.92 ± 0.41	7.22 ± 0.34	94.00 ± 4.43
P2.5/C7.5	2.5	7.5	10.0	61.14 ± 4.44	1.77 ± 0.87	63.32 ± 3.39
P5/C7.5	5.0	7.5	12.5	66.54 ± 4.74	3.08 ± 0.15	65.54 ± 3.05
P2.5/C15	2.5	15.0	17.5	67.06 ± 5.08	2.03 ± 0.14	69.87 ± 4.65
P5/C15	5.0	15.0	20.0	79.91 ± 5.01	3.52 ± 0.20	70.12 ± 4.07

* Data are shown as the means ± standard deviation, *n* = 30. ** Data are shown as the means ± standard deviation, *n* = 3.
